# Theranostic Applications of Scaffolds in Current Biomedical Research

**DOI:** 10.7759/cureus.71694

**Published:** 2024-10-17

**Authors:** Sarika J Patil, Vandana M Thorat, Akshada A Koparde, Rohit R Bhosale, Somnath D Bhinge, Dhanashri D Chavan, Devkumar D Tiwari

**Affiliations:** 1 Department of Pharmacology, Krishna Institute of Medical Sciences, Krishna Vishwa Vidyapeeth (Deemed to be University), Karad, IND; 2 Department of Pharmaceutical Chemistry, Krishna Institute of Pharmacy, Krishna Vishwa Vidyapeeth (Deemed to be University), Karad, IND; 3 Department of Pharmaceutics, Krishna Foundation's Jaywant Institute of Pharmacy, Karad, IND; 4 Department of Pharmaceutical Chemistry, Rajarambapu College of Pharmacy, Kasegaon, IND

**Keywords:** 3d printing, patient outcome, regenerative medicine, scaffold, smart drug delivery, theranostic, tissue engineering

## Abstract

Theranostics, a remarkable combination of diagnostics and therapeutics, has given rise to tissue/organ-format theranostic scaffolds that integrate targeted therapy and real-time disease monitoring. The scaffold is a 3D structuring template for cell or tissue attachment and growth. These scaffolds offer unprecedented opportunities for personalized medicine and hold great potential for revolutionizing healthcare. Recent advancements in fabrication techniques have enabled the creation of highly intricate and precisely engineered scaffolds with controllable physical and chemical properties, enhancing their therapeutic potential for tissue engineering and regenerative medicine. This paper proposes a new categorization method for scaffolds in tissue engineering based on the relativity of scaffold design-independent parameters. Five types of scaffolds are defined at different levels, highlighting the importance of understanding and analyzing scaffold types. It possesses the ability to seamlessly integrate diagnostics and therapeutics within a single platform, enhancing the efficacy and precision of personalized medicine. Natural scaffolds derived from biomaterials and synthetic scaffolds fabricated by human intervention are discussed, with synthetic scaffolds offering advantages such as tunable mechanical properties and controlled drug delivery, while natural scaffolds provide inherent biocompatibility and bioactivity, making them ideal for promoting cellular responses. The use of synthetic scaffolds shows great promise in advancing regenerative medicine and improving patient outcomes. The transfer of new technologies and changes in society have accelerated the evolution of health monitoring into the era of personal health monitoring. Using emerging health data, cost-effective analytics, wireless sensor networks, mobile smartphones, and easy internet access, the combination of these technologies is expected to accelerate the transition to personal health monitoring outside of traditional healthcare settings. The main objective of this review article is to provide a comprehensive overview of the theranostic applications of scaffolds in current biomedical research, highlighting their dual role in therapy and diagnostics. The review aims to explore the latest advancements in scaffold design, fabrication, and functionalization, emphasizing how these innovations contribute to improved therapeutic efficacy, targeted drug delivery, and the real-time monitoring of disease progression across various medical fields.

## Introduction and background

**Figure 1:**
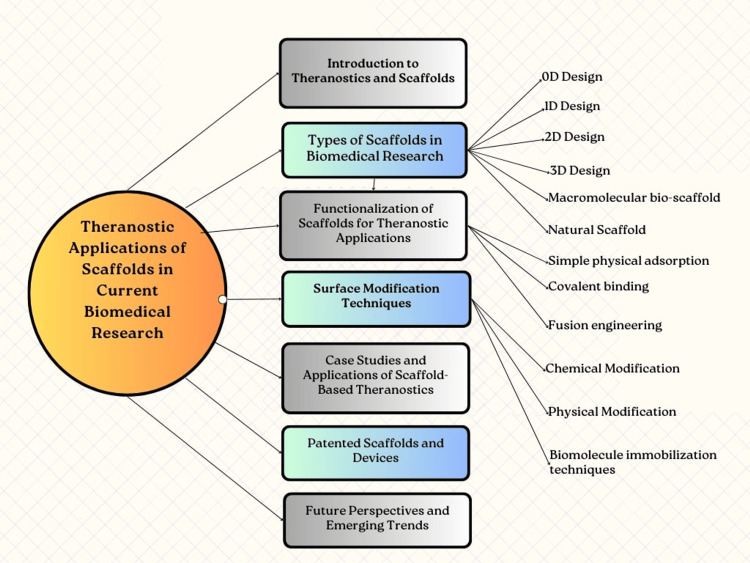
Pictorial representation of the review points

Theranostics, a remarkable combination of the diagnostics and therapeutics of disease on a single platform, has emerged as a highly promising field for the development of innovative personalized medicine. Over the past few decades, there have been extensive and rigorous efforts to develop a wide range of theranostic agents and scaffolds. These agents and scaffolds enable magnetic resonance imaging, fluorescence imaging, computed tomography, and photoacoustic imaging while simultaneously facilitating photodynamic/photothermal chemotherapy, chemotherapy drug delivery, radiotherapy, and many more. Their multifunctionality stems from their composition, which often incorporates bioactive materials or functional polymers that achieve a certain degree of bioactivity and versatility [1,2]. However, it is worth noting that the majority of theranostic agents and scaffolds currently available have focused primarily on achieving a certain level of functionality in terms of bioactivity and versatility, without substantial consideration for tissue/organ-specific theranostic applications. The continuous progress in our understanding of the intricate interplay and synergistic effects between disease pathology, tissue architectures, and therapeutic activities has paved the way for the development of advanced theranostic scaffolds [3,4]. These scaffolds not only allow for monitored diagnostics but also prompt therapeutics by providing an organized and porous tissue-supporting environment. In the context of biomedical research, these novel tissue/organ-format theranostic scaffolds are emerging as ideal candidates. They possess the ability to seamlessly integrate diagnostics and therapeutics within a single platform, enhancing the efficacy and precision of personalized medicine. By leveraging the unique properties of their organized porous tissue-supporting environment, these theranostic scaffolds offer unparalleled opportunities for targeted therapy and the real-time monitoring of the disease [5,6]. With the advent of tissue/organ-format theranostic scaffolds, researchers and clinicians now have an unprecedented toolkit to combat diseases effectively [7]. As we continue to delve deeper into the complexities of disease pathology and tissue architecture, the future holds great promise for the realization of increasingly sophisticated and tailored theranostic approaches [8,9]. The unique combination of controllable physical and chemical properties, as well as the ability to create precise three-dimensional (3D) structures, makes organized porous scaffolds an excellent option for supporting the growth and organization of different cells for personalized medicine [10]. These scaffolds have been designed with a variety of features that allow for the engineering of tissue formations and the mimicking of tissue injuries, all of which can be tailored to meet the needs of individual patients. Recent advancements in fabrication techniques, such as nano-sculpturing which is a technique used to tailor the physical, chemical, and biological properties of materials, often for applications in areas like electronics, photonics, and biomedicine, and the tailored fabrication of synthetic materials, have expanded the range of scaffolds available for therapeutic applications [11,12]. Furthermore, the use of state-of-the-art technologies like 3D printing and nanotechnology has made it possible to create highly intricate and precisely engineered scaffolds. This level of control over the physical and chemical properties of the scaffold has opened up new possibilities for personalized medicine, as it allows for the creation of tissue constructs that closely resemble native tissues. The development of advanced fabrication techniques, such as electrospinning and microfluidics, improves scaffold functionality by enabling better cell interaction, targeted drug delivery, and tissue regeneration. It has also revolutionized the production of micro-/nano-scaled scaffolds, providing precise control over their morphology and structure [13,14]. Additionally, the incorporation of bioactive molecules and growth factors has enhanced the therapeutic potential of these scaffolds, making them suitable for targeted drug delivery and tissue regeneration. Munot et al. have formulated and evaluated chitosan-poly(lactic-co-glycolic acid) (PLGA) biocomposite scaffolds incorporated with bioactive quercetin liposomes prepared by quality-by-design techniques for the improved healing of oral lesions [15]. In conclusion, organized porous scaffolds are a versatile and promising platform for tissue engineering and regenerative medicine, with the potential to significantly improve patient outcomes and provide transformative medical solutions [16-19]. Figure 1 depicts a pictorial representation of this review article's points.

Methods

Literature Search Strategy

A comprehensive literature search was conducted to gather relevant articles on the theranostic applications of scaffolds in biomedical research. The databases utilized for the search included PubMed, Google Scholar, and Scopus. The search covered a 20-year period, from 2003 to 2023, to capture the most recent advances in the field. Key search terms used were as follows: theranostics, scaffolds, biomaterials, drug delivery, imaging, nanotechnology, and biomedical applications. These keywords were combined using Boolean operators (AND, OR) to ensure a broad and inclusive search. For instance, searches such as "theranostics AND scaffolds" or "scaffolds AND drug delivery AND imaging" were performed to identify relevant articles.

Inclusion and Exclusion Criteria

The following criteria were applied to select articles for this review:

Inclusion criteria: Studies that focused on the dual diagnostic and therapeutic applications (theranostics) of scaffolds, articles published in peer-reviewed journals, studies highlighting the biomedical applications of scaffolds in areas such as drug delivery, tissue engineering, and disease diagnostics, and both experimental studies and comprehensive reviews were included.

Exclusion criteria: Articles not focused on theranostics (i.e., studies on scaffolds only used for structural or therapeutic purposes without diagnostic relevance), studies outside the biomedical scope (e.g., articles focusing on industrial or non-medical uses of scaffolds), articles published before 2013, unless they provided seminal insights relevant to the evolution of the field, and non-English language publications were excluded.

Selection Process

After conducting the search, the titles and abstracts of the articles were screened for relevance. Full-text articles were then reviewed to ensure they met the inclusion criteria. Duplicate articles and those lacking sufficient relevance to theranostic scaffold applications were excluded. Cross-referencing was also performed, where key references from selected papers were reviewed to ensure comprehensive coverage of the field.

Final article count: Following this process, a total of 100-120 articles were selected for inclusion in this review. These articles represent a diverse range of studies, including experimental research, clinical trials, and critical reviews, providing a holistic overview of current trends and advancements in theranostic scaffolds in biomedical research.

## Review

Types of scaffolds in biomedical research

**Figure 2:**
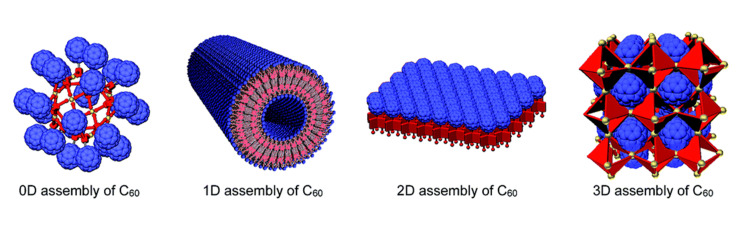
Different types of scaffolds Image Credit: Ishiwari et al., 2018 [20]; published with permission from the Royal Society of Chemistry 0D: zero-dimensional: 1D: one-dimensional; 2D: two-dimensional; 3D: three-dimensional

The interpretation of a scaffold is, however, a conceptual and subjective matter. In order to facilitate a better understanding and scientific study of scaffolds in tissue engineering, a new categorization method is proposed here. This method introduces the notion of the relativity of scaffold design-independent parameters. By considering these parameters, a taxonomy of scaffolds is presented at five defining levels [20]. The detailed structure of the scaffolds is depicted in Figure 2.

Scaffold Number 1

It has a mostly zero-dimensional (0D) design. These tiny structures play a crucial role in assembling functional molecular units into specific arrangements. These scaffolds are spherical assemblies that serve as platforms for organizing functional molecular units. Functional molecules can be either incorporated inside the scaffold or attached to its outer surface.

Applications: There are several applications of 0D supramolecular scaffolds. The first is efficient light harvesting, which enhances light absorption and energy transfer. The second is multivalent molecular recognition, which is useful for binding multiple ligands. The third is catalytic systems, which facilitate chemical reactions. The last is templates for inorganic material synthesis, which assist in creating discrete inorganic structures.

Design and exploration*: *By strategically attaching functional units to these scaffolds, we can achieve specific structures. These structures enable the exploration of novel properties beyond what individual molecular units can achieve alone. In summary, 0D supramolecular scaffolds provide a fascinating avenue for designing organic and polymeric materials [21].

*Scaffold Number 2* 

It is with a one-dimensional (1D) design. These scaffolds play a crucial role in providing a supportive environment for cell growth and tissue regeneration. 1D scaffolds are linear structures that serve as templates for tissue development. They can be fibers, nanotubes, or other elongated forms like nanowires, rod-like particles, and microfilaments.

Applications:Several applications of 1D scaffolds include the following: The first is nerve regeneration. In nerve tissue engineering, aligned 1D scaffolds guide axon growth. The second is vascular tissue engineering, where 1D scaffolds mimic blood vessels and promote endothelial cell attachment. The third is muscle repair, where muscle fibers can regenerate along 1D scaffolds. Materials used to prepare 1D scaffolds are natural polymers, and examples include collagen, silk, and fibrin. The fourth is synthetic polymers, comprising poly(lactic acid) (PLA), poly(glycolic acid) (PGA), and their copolymers. The last is carbon nanotubes which are used for electrical conductivity and mechanical reinforcement.

Fabrication techniques*: *Fabrication techniques of 1D scaffolds are as follows: electrospinning, which produces nanofiber scaffolds; template-assisted synthesis, which uses sacrificial templates to create 1D structures; and self-assembly or molecular self-organization into fibers, where characterization is done by assessing fiber diameter, alignment, and mechanical properties and evaluating cell attachment and proliferation on 1D scaffolds [22].

Scaffold Number 3 

It is with a two-dimensional (2D) design. It refers to a 2D representation of a scaffold structure. Scaffolds may have non-hazardous properties such as biocompatibility and biodegradability for the human body. Mechanical properties must support body weight or perform other roles based on the type of tissue. Optimized scaffold structures, including porosity, pore size, and pore shape, promote cell viability and proliferation. Conventional fabrication methods include thermally induced phase separation, emulsion freeze-drying, solvent casting, gas forming, and electrospinning. 3D printing is particularly suitable for bone tissue engineering due to its ability to manufacture complex structures. Biomaterials fall into four categories: polymer, ceramic, metal, and composites. Composites blend two or more biomaterials to achieve desired properties tailored to individual patient conditions. Balancing fabrication methods with biomaterial selection is crucial for successful bone tissue engineering [23].

Scaffold Number 4

There are some inherent dynamic functions or 3D scaffold products.

3D scaffolds in tissue engineering: 3D scaffolds provide an environment conducive to integrating cells or growth factors for tissue repair or replacement. Cellular response and fate depend on scaffold material properties. The porous, networked structure allows nutrient, oxygen, and waste exchange. Various methods create 3D scaffolds with variable pore sizes and porosities, including conventional and rapid prototyping techniques.

Graded porosity: Mimicking in vivo situations, graded porosity can expose cells to layers with changing characteristics. Investigating extracellular matrix (ECM) properties and mechanical effects is crucial for scaffold design. Exciting alternative techniques like 3D bioprinting, hydrogels, and nanofibers offer promising avenues. Creating graded porosity in tissue engineering scaffolds presents several challenges like achieving uniform pore distribution, controlling pore size at different scaffold regions, ensuring mechanical stability while maintaining porosity, and replicating the complex microenvironment of natural tissues. These factors are critical for successful cell infiltration, nutrient diffusion, and tissue regeneration.

Design complexity*: *Achieving a gradual transition from one porosity level to another requires intricate design. Balancing structural integrity, mechanical properties, and biological functionality is challenging. Integrating a gradual porosity transition in scaffolds requires striking a delicate balance between mechanical strength, biological compatibility, and fabrication precision, which makes the process technically demanding.

Fabrication techniques:Fabricating graded porosity demands precise control over pore size and distribution. However, precise control over pore size and distribution ensures the scaffold's functionality, structural integrity, and ability to support natural tissue growth. Conventional methods (e.g., freeze-drying, solvent casting) may not achieve the desired gradient.

Material compatibility*: *Different materials with varying degradation rates must harmonize. Harmonizing materials with varying degradation rates is crucial for ensuring that the scaffold effectively supports tissue regeneration, maintains mechanical integrity, promotes favorable cellular responses, and minimizes adverse reactions. This thoughtful integration ultimately leads to improved outcomes in tissue engineering applications. Ensuring compatibility between adjacent layers is crucial for tissue integration.

Cell behavior*: *Cells respond differently to varying porosities. Understanding cell migration, adhesion, and differentiation within the gradient is essential.

Biological functionality: Graded porosity should enhance nutrient diffusion, waste removal, and cell signaling. Ensuring optimal biological function across the scaffold is a significant challenge, which may be one of the prime reasons [24].

*Scaffold Number 5* 

It is a fully dynamic macromolecular bio-scaffold system. Scaffold proteins serve as molecular platforms to bring together many different types of partner proteins. They are the building blocks for efficient signal cascades. Over 300 human proteins serve as scaffolds in signal transduction pathways. Recognized as static platforms, scaffolds possess dynamic behavior. These structural variations affect their signal transduction pathways. An example of scaffolds that at least in some cases display conformational changes would be the yeast scaffold STE5 or human scaffolds such as kinase suppressor of Ras (KSR), nuclear factor kappa B (NF-κB) essential modulator (NEMO), and SH3 and multiple ankyrin repeat domains 3 (SHANK3). KSR is a scaffold protein crucial for the Ras signaling pathway, facilitating interactions among components of the mitogen-activated protein kinase (MAPK) signaling cascade to propagate signals from growth factors and extracellular stimuli. NEMO is essential for the NF-κB signaling pathway, regulating immune responses, inflammation, and cell survival by acting as a scaffold that brings together various signaling molecules, while SHANK3 is predominantly located in the postsynaptic density of neurons, where it links neurotransmitter receptors to intracellular pathways and organizes signaling complexes critical for synaptic function and plasticity. Both traits are also related to the dynamicity of scaffolds. The clinical implications are significant, as it is conceivable that abnormal conformational changes in scaffolds may contribute to the pathogenesis of various diseases. Knowing more about these dynamics will help us develop better treatments for scaffold-associated disorders. To summarize, scaffold proteins are the crux in making signaling cascades more sensitive and form as a switchboard that orchestrates different cellular processes. Their flexibility and adaptability play critical roles in cellular communication [15,25,26]. It is important to note that these five types of scaffolds differ from one another in a relative approach.

Moreover, the meanings of various published scaffold terms are also interpreted and elucidated based on this new scaffold classification. Through this interpretation, the importance and advantages of this systematic classification method are conclusively highlighted. The significance of this categorization method lies in its ability to provide valuable insights and a comprehensive framework for better understanding and analyzing different types of scaffolds in tissue engineering. Consequently, this establishes a robust foundation for future research and development in the field, ultimately resulting in improved therapeutic outcomes and advancements in regenerative medicine. By embracing this new categorization method, the scientific community can unlock new perspectives and possibilities in the realm of tissue engineering and scaffold design [27-31].

Biomedical scaffolds are generally categorized as either natural scaffolds that are derived from biomaterials called decellularized ECM (dECM) obtained from natural sources through chemical and biological extractions or synthetic scaffolds that are designed and fabricated by human intervention and usually constituted of materials such as polycaprolactone (PCL) and PLA [32]. Natural scaffolds have accessible knot adhesion sites that may facilitate cell anchorage on the scaffold, and these scaffolds resemble the natural tissue ECM in relation to their biochemical and structural properties; however, these scaffolds have high batch-to-batch variability and are immunogenic due to the presence of xenogeneic proteins [33,34]. Synthetic scaffolds are more attractive in tissue engineering applications since they are more adaptable compared to natural scaffolds and they can be tailor-made with predetermined physicochemical properties and are expected to have lower batch-to-batch variability in bioprocessing [35,36]. Furthermore, synthetic scaffolds provide a versatile platform for controlled drug delivery, allowing for a localized and sustained release of therapeutic agents. This capability opens up new possibilities in regenerative medicine, where specific growth factors or drugs can be incorporated into the scaffold matrix to promote tissue regeneration and repair [37,38]. In addition, synthetic scaffolds offer the advantage of tunable mechanical properties, which can be optimized to mimic the target tissue's stiffness and elasticity. This is crucial for successful tissue engineering, as the mechanical cues provided by the scaffold can influence cell behavior and tissue development [39]. Moreover, synthetic scaffolds can be fabricated with precise spatial organization, allowing for the creation of complex 3D structures that closely resemble the architecture of native tissues. This enables the development of more realistic in vitro* *models for studying disease progression and drug testing [40]. Overall, the use of synthetic scaffolds in tissue engineering holds great promise for advancing regenerative medicine and improving patient outcomes also [41,42].

Natural scaffolds

Natural scaffolds have attracted extensive attention for theranostic applications in biomedical research due to their remarkable biocompatibility and highly beneficial bioactive properties. These incredible structures, known as natural scaffolds, encompass various substances such as ECM proteins and polysaccharides [43,44]. They form a multifaceted, intricate 3D cage that gently envelops tissues and organs, ultimately providing the perfect cell micro-architecture necessary for optimal cellular functions and overall health [45]. It is worth noting that natural scaffolds not only provide structural solidity but also emit crucial bioactive signals that orchestrate specific cellular responses in a well-controlled, spatiotemporally precise manner. These responses encompass a wide range of cellular behaviors, including attachment, apoptosis, proliferation, signaling, and differentiation, among others [46,47]. This remarkable phenomenon makes natural scaffolds an invaluable tool in various therapeutic applications such as tissue engineering, drug delivery, and regenerative medicine [48,49].

Moreover, natural scaffolds possess the remarkable ability to modify their structure effortlessly, empowering them to embark on sensing, imaging, and diagnostic endeavors. By harnessing the capabilities of these natural scaffolds, the medical field can achieve an extraordinary fusion of imaging techniques and targeted treatments specifically tailored to different species. This approach holds incredible promise for the future of personalized medicine, opening up new horizons for improved patient care and treatment outcomes [50-53]. ECM is composed of a complex network of proteins and polysaccharides, playing a pivotal role in providing not only robust support for the cells but also intricate biochemical and mechanical cues for precisely regulating cell behaviors and tissue functions [54]. In the realm of tissue engineering and regenerative medicine, tremendous efforts have been dedicated to exploring the vast potential of natural ECM materials, including collagen, gelatin, and various bioactive polymer scaffolds. Remarkably, these ECM-derived scaffolds exemplify great resemblance to the native tissue properties, encompassing their biochemical composition, architectural arrangement, viscoelasticity, and inherent biological cues [55-57]. As a result, they have emerged as formidable contenders for the development of advanced in vivo theranostic platforms, revolutionizing the possibilities for regenerative therapies and diagnostics [58]. Furthermore, expanding the horizons of theranostics, naturally derived polysaccharide scaffolds, such as hyaluronic acid, alginate, chitosan, and chitin, have also showcased their promising prospects in the field of tissue engineering, further amplifying their potential applications in therapeutics, diagnostics, and beyond [59-61].

The symbiotic integration of these natural ECM-derived materials and polysaccharide scaffolds heralds a new era in healthcare, where the boundaries of tissue regeneration, disease diagnostics, and targeted therapeutics are continuously pushed and surpassed [62-64]. Engineered scaffolds have been proven as an effective platform for biomedical applications. Strong scaffold design consideration is needed for their applications. It is crucial to consider the desired permeability, elasticity, degradation, bioactivity, biocompatibility, and fabrication for scaffolds [65,66]. All of these properties may affect the therapeutic outcomes from a scaffold in tissue engineering or drug delivery applications since the diffusion rate of cells or drugs is linked to pore structure (size, shape, and connectivity) and permeability [67]. It is generally preferred to have a porous scaffold with a porosity greater than 90% and with interconnected pores ranging from 100 to 500 μm in diameter to achieve good cell infiltration [68-70]. Furthermore, the proper consideration of pore structure and permeability plays a significant role in ensuring the success of scaffold-based therapies [71,72]. In addition to pore structure and permeability, elasticity, which is closely connected to mechanical compliance, affects the retention ability of either the cells in tissue regeneration or the compounds in drug delivery applications. Scaffolds being too soft or too rigid may result in inefficient tissue formation or disease recurrence. Thus, an optimal scaffold design should strike a balance between mechanical strength and flexibility to achieve the desired therapeutic outcomes. By carefully adjusting the scaffold's elasticity, it becomes possible to create an environment that promotes cell adhesion, migration, and differentiation. Moreover, the inclusion of appropriate bioactive molecules within the scaffold can further enhance cell functionality and tissue regeneration processes [73-75].

Fabrication techniques also play a crucial role in scaffold design. Depending on the application, different fabrication approaches such as electrospinning, 3D printing, or freeze-drying can be utilized to achieve the desired scaffold architecture [76]. The choice of fabrication method should consider not only the structural requirements but also the scalability, reproducibility, and cost-effectiveness of the process. With advancements in technology, novel fabrication techniques are continually being developed, enabling the production of scaffolds with tailored properties and complex geometries [77-80]. Overall, the design of engineered scaffolds for biomedical applications requires careful consideration of various factors, including permeability, elasticity, degradation, bioactivity, biocompatibility, and fabrication [81,82]. By optimizing these properties, it is possible to tailor the scaffold to specific therapeutic needs, improving the efficacy of tissue engineering and drug delivery approaches. Continuous research and innovation in scaffold design will pave the way for further advancements in regenerative medicine and patient care [83-87]. The high biocompatibility of scaffolds is vital to avoid adverse immune responses. Good bioactivity helps the parent scaffolds promote cell adhesion, migration, proliferation, and influx. Proper degradation is necessary to provide space for newly formed tissue and to achieve the gradual release of therapeutic agents in a sustained manner. It is also desired for a biodegradable scaffold to be low-cost, eco-friendly, and easily fabricated [88-90]. Finally, safety and reproducibility must be ensured in scaled-up scaffolds for clinical applications. Overall, the development of biomaterials for scaffolds with a wide range of functions requires interdisciplinary efforts and continuous innovations [91-95].

Functionalization of scaffolds for theranostic applications

Given the innate advantages of scaffolds in providing a favorable and interactive microenvironment for cells, diverse strategies have been extensively explored to further enhance their therapeutic efficacy and diagnostic sensitivity by "functionalization" [96]. These strategies include simple physical adsorption, covalent binding, and fusion engineering, which are all versatile methods for powering scaffolds with distinctive and synergistic features. The contemporary advances of scaffolds in the fields of oncology, orthopedics, and tissue engineering have been significantly propelled by substantial endeavors in drug delivery, imaging, and combinatorial applications [97-99]. In the realm of drug delivery, scaffolds have been engineered to precisely control the release of therapeutics, ensuring the optimal dosage and duration of treatment. Furthermore, the integration of imaging agents into scaffolds enables the real-time monitoring of cellular behavior and therapeutic response, allowing for personalized and targeted interventions [100-102]. Additionally, the combinatorial applications of scaffolds with other innovative technologies, such as 3D printing and nanotechnology, have revolutionized tissue engineering, enabling the fabrication of highly complex and functional constructs with enhanced regenerative capabilities.

Collectively, these advancements highlight the immense potential of functionalized scaffolds in revolutionizing healthcare and biomedical research, paving the way for the development of novel and more effective treatment options for a wide range of diseases and clinical conditions [103,104]. In the context of tumor therapy and bone regeneration, poly(L-lactic acid) (PLLA)/nanohydroxyapatite (nHA) scaffolds have been fabricated to load the anti-cancer drug microtubule-stabilizing agent (paclitaxel or docetaxel and mesenchymal-epithelial transition (MET)). The findings indicate that MET could both kill tumor cells by inducing cell cycle arrest and apoptosis and accelerate the osteogenic differentiation of human bone marrow mesenchymal stem cells (hBMSCs) by activating the Wnt/β-catenin pathway. Due to their high specific surface area and porous structure, PLLA/nHA scaffolds could load a high dose of MET (≈41% in weight) for sustained release (up to 35 days).* *In vitro release results showed that the release of MET exhibited a burst on the first day (≈12%) and continued to release for 35 days. The addition of MET significantly promoted the early stage of alkaline phosphatase (ALP) activity of hBMSCs by 3.2 times (the 14th day) compared to control scaffolds without MET, whereas control scaffolds failed to promote the osteogenic differentiation of hBMSCs. On the 28th day, the mineralization of hBMSCs on MET-loaded scaffolds (PLLA/nHA/MET) was significantly enhanced, which further validated the pro-osteogenic effect of MET [105]. Core/shell mesoporous silica nanostructures have emerged as an exquisite nanoplatform for theragnostic applications due to their unique properties of bio-mineralized surface, tunable mesopore size, exceptional surface area (americium up to 2500 m^2^/g), and high biodegradability and biocompatibility. These nanostructures have been surface-modified using various targeting moieties such as folic acid, antibodies, polysaccharides, aptamers, polymers, and proteins establishing themselves as novel platforms for specific cancer targeting. A stable mesoporous silica nanoparticle structure enables the selective incorporation of imaging probes (magnetic resonance imaging contrast agents, quantum dots, dyes, radionuclides, and metals) and nanoparticles (gold, magnetic, etc.) in either the core or shell leading to a combined system for multimodal imaging [106].

Surface modification techniques

Scaffolds, which are artificial structures made of synthetic or natural biomaterials, support the growth and attachment of cells, tissues, and organs. They mimic the properties of the ECM of a specific tissue and can regenerate, repair, or replace damaged tissues. Scaffolds can be fabricated using various techniques that define specific final properties, such as mechanical, physical, or biological. Furthermore, scaffolds can also be modified by strategies such as chemical, physical, or biomolecule immobilization to improve their biocompatibility and mechanical properties. This is often the first step in enhancing the functionality of the scaffolds for desired biomedical applications [107]. Surface modification techniques are necessary for improving the scaffold's biocompatibility and mechanical properties and disposing of functionalities on the scaffolds for desired applications. Surface modification techniques may be of various types such as (1) chemical modification, (2) physical modification, and (3) biomolecule immobilization.

Natural and synthetic biomaterials like metals, ceramics, polymers, and composites are used as scaffolds, influencing cell attachment, growth, proliferation, and differentiation. Stiffness, roughness, surface energy, pore size, and layer thickness are some parameters that impact the scaffold's biocompatibility and functionality [108-111]. To modify a scaffold's surface passively, typically by adjusting its properties, often requires sophisticated and expensive processing conditions. Surface modification techniques via manipulating physical or chemical properties have been developed to enhance the biocompatibility and functionality of scaffolds for biomedically desired applicability [112]. In recent years, there has been a significant advancement in surface modification techniques, driven by the ever-growing demand for advanced biomedical applications. These techniques have not only allowed for the improvement of biocompatibility but have also paved the way for tailoring the mechanical properties of scaffolds to cater to specific requirements. The field of surface modification encompasses a wide range of strategies, each playing a crucial role in enhancing scaffold functionality [113]. Chemical modification stands out as one of the key techniques employed in surface modification. This approach involves altering the scaffold's chemical composition through various methods, such as the introduction of functional groups or the application of surface coatings. By carefully manipulating the chemical properties, researchers can achieve remarkable enhancements in the biocompatibility and functionality of scaffolds, opening up new avenues for innovative biomedical applications. Physical modification techniques offer another means to optimize scaffold performance.

These methods focus on altering the scaffold's physical attributes, including its topography, roughness, and pore size. By precisely controlling these parameters, researchers can create surfaces that guide cellular behavior and promote favorable cell-scaffold interactions. Furthermore, the use of advanced fabrication techniques allows for the development of 3D structures with intricate surface patterns, mimicking natural ECM and facilitating cellular integration [114]. In addition to chemical and physical modifications, biomolecule immobilization techniques have gained significant attention for their ability to enhance scaffold biocompatibility. By immobilizing bioactive molecules, such as growth factors or adhesion peptides, onto the scaffold surface, researchers can create an environment that promotes cell attachment, growth, and proliferation. This approach not only mimics the natural cellular microenvironment but also enables precise control over cellular responses, making it highly beneficial for tissue engineering and regenerative medicine applications. It is important to note that the choice of scaffold material plays a fundamental role in determining its biocompatibility and functionality. Different materials, ranging from metals and ceramics to polymers and their composites, exhibit unique properties and structures that directly influence cellular behavior. Researchers continue to explore novel materials and fabrication techniques to develop scaffolds that offer optimal performance for specific biomedical applications [115]. In conclusion, surface modification techniques have revolutionized the field of scaffold engineering, enabling the development of tailored structures with enhanced biocompatibility and functionality. The constant advancements in chemical, physical, and biomolecule immobilization approaches hold great promise for the future of biomedical applications, facilitating breakthroughs in tissue engineering, regenerative medicine, and beyond. With further research and innovation, surface modification techniques will continue to drive the progress of biomedical technologies, ultimately improving the quality of life of countless individuals around the world [116,117].

Case studies and applications of scaffold-based theranostics

Although recent advances in the development, design, and fabrication of tissue scaffolds have resulted in significant improvements in matching scaffold architecture and composition to host tissue contradictions, a wide range of challenges still remain in this field. These challenges significantly impede the realization of fully integrated design approaches for the optimization of scaffolds tailored specifically to each patient's unique application. Furthermore, it is crucial to maintain active research efforts at the forefront of scaffold design for medical applications. This continuous progress will ensure that medical doctors retain the utmost confidence in the efficacy and reliability of tissue scaffolds when utilized in in vivo applications. Recently, there have been notable advancements in the design, fabrication, and assessment of tissue scaffolds. These advancements provide a robust foundation on which to build towards further improvements in the matching of porous scaffold materials to the physiological environment and the required biological function. This alignment between the scaffold and the surrounding physiological conditions is critical for achieving optimal tissue regeneration outcomes. By capitalizing on the latest research findings, scientists and engineers can work towards refining scaffold design parameters, such as pore size, porosity, mechanical properties, and degradation rates. Tailoring these parameters intricately to match the complex and diverse needs of different tissues will undoubtedly push the boundaries of tissue engineering and regenerative medicine to new heights. Additionally, closer collaboration between multidisciplinary teams, including researchers, clinicians, and materials scientists, is essential in addressing the existing challenges comprehensively. Such collective efforts will foster innovative strategies for scaffold fabrication, the incorporation of bioactive molecules, and the enhancement of scaffold-host tissue interactions. Moreover, additional research should be focused on the long-term performance and biocompatibility of tissue scaffolds. This emphasis will enable researchers to gain deeper insights into the scaffold-host tissue interface, thereby promoting the development of improved strategies and materials for long-lasting and successful tissue regeneration.

In summary, continuous advancements in the development, design, and fabrication of tissue scaffolds provide a promising outlook in the field of regenerative medicine. By addressing the existing challenges and building upon the current foundation, scientists and clinicians can collaborate to unlock the full potential of tissue engineering, ultimately revolutionizing healthcare and benefiting countless patients worldwide [118]. The complexities of tissue scaffold applications often invoke thought across a number of disciplines, each of which will introduce conditions that must be satisfied for positive outcomes. As tissue scaffolds can be a passive addition to a treatment or intervention, robust design methodologies and simulations should guide their use; however, active considerations or intricate processes may be beyond the scope of computational modeling methodologies. Nevertheless, understanding scaffold design, fabrication, and assessment procedures is vital knowledge for researchers to successfully implement scaffolds in their own work, as all three processes can magically influence outcomes [119]. Some patented scaffolds and devices are mentioned in Table 1.

**Table 1:** List of patented scaffolds 3D: three-dimensional; Fc: fragment crystallizable; MAC: methyl acrylate gelatin; MACh: methacrylate chitosan; HA: hydroxyapatite; TCP: tricalcium phosphate; Smurf1: Smad ubiquitination regulatory factor 1; PLA: poly(lactic acid); PLGA: poly(lactic-co-glycolic acid)

Sr. no.	Patent name	Patent number	Inventor	Device or system developed	Description	Year	Reference
1	Polymer-based oxygen sensors	CA2904127C (Canada)	Soya Gamsey, Natalie Wisniewski, Kristen Helton, William Mcmillan	Device	The disclosure describes sensors that detect substances using a polymer with a stable luminescent porphyrin dye, which works in the near-infrared range and is suitable for mammalian skin	Granted: 2023-10-17. Published: 2023-10-17	[120]
2	Systems, apparatus and methods for cryogenic 3D printing	US11584066B2 (United States)	Boris Rubinsky, Michal Adamkievicz, Ze'ev Shaked	Device with method	This technology uses cryogenic 3D printing to create precise micro- and macrostructures for tissue engineering, drug delivery, and the food industry. By immersing the printed object in a liquid coolant, it precisely controls the freezing process, managing temperature, flow rate, and fluid volume for consistent frozen layers	Granted: 2023-02-21. Published: 2023-02-21	[121]
3	Biocompatible and conductive hydrogels with tunable physical and electrical properties	US11028211B2 (United States)	Nasim Annabi, Iman Noshadi	Drug system	This technology provides a biodegradable, biocompatible hydrogel with adjustable conductivity. By altering the polymer-to-bio-ionic liquid ratio or the weight percentage of the conjugated polymer, its mechanical and electrical properties can be controlled. The preparation method is also detailed	Granted: 2021-06-08. Published: 2021-06-08	[122]
4	Silk-based scaffold platform for engineering tissue constructs	US10058514B2 (United States)	Lindsay Wray, Jelena Rnjak-Kovacina, David L. Kaplan	Drug system	These inventions describe silk-based scaffolds for tissue engineering, featuring tunable properties like porosity, mechanical strength, and degradation rate. They can include hollow conduits for nutrient and oxygen delivery, aiding in the creation of complex tissue equivalents	Granted: 2018-08-28. Published: 2018-08-28	[123]
5	Dynamic silk coatings for implantable devices	US11266339B2 (United States)	David L. Kaplan, Lee W. Tien, Gary G. Leisk, Tim Jia-Ching Lo, Cinzia Metallo, Fiorenzo Omenetto	Device and drug system	This describes implantable devices with dynamic silk coatings that can be formed directly in the body (in situ) or within a living organism (in vivo)	Granted: 2022-03-08. Published: 2022-03-08	[124]
6	Biomedical patches with aligned fibers	US20220175510A1 (United States)	Matthew R. MacEwan, Jingwei Xie, Zack Ray, Younan Xia	Drug system	This method creates aligned fibrous structures using an electrode-based system. It involves positioning a second electrode within an area defined by the first electrodes, which may have rounded surfaces and be arranged in an array. This setup enables the formation of fibrous structures	Published: 2022-06-09	[125]
7	Resorbable ceramics with controlled strength loss rates	US9795716B2 (United States)	Susmita Bose, Amit Bandyopadhyay	Drug system	This describes bio-resorbable and biocompatible compositions for tissue or bone regeneration. The material is a calcium phosphate-based ceramic scaffold, which can be porous or non-porous and contains metal ion or metal oxide dopants. It is suitable for cell or tissue scaffolds and resorbs at a controlled rate, gradually losing strength under body conditions	Granted: 2017-10-24. Published: 2017-10-24	[126]
8	Method for manufacturing a porous ceramic scaffold having an organic/inorganic hybrid coating layer containing a bioactive factor	US8734831B2 (United States)	Hyoun-Ee Kim, Shin-Hee Jun, Eun-Jung Lee	Drug system	This method involves creating a porous ceramic scaffold, mixing silica xerogel with an organic substance to form a hybrid composite solution, adding a bioactive factor, and coating the scaffold. The scaffold maintains open pores and gradually releases the bioactive factor over time	Granted: 2014-05-27. Published: 2014-05-27	[127]
9	In vivo live 3D printing of regenerative bone healing scaffolds for rapid fracture healing	US11642849B2 (United States)	Venu G. Varanasi, Azhar Ilyas, Philip Roger Kramer, Taha Azimaie, Pranesh B. Aswath, Tugba Cebe	Drug system	Bio-inks are used for 3D tissue repair and regeneration, forming biodegradable scaffolds at specific sites. Specific hydrogels include MAC and MACh, which can be formulated with sucrose, a silicate component (e.g., laponite), or a cross-linking agent. Kits for point-of-care tissue repair demonstrate superior tissue regeneration (up to 99.85% in four weeks) and rapid in situ tissue repair and bone formation	Granted: 2023-05-09. Published: 2023-05-09	[128]
10	Stem cells and devices for bone regeneration	US11484625B2 (United States)	Yang Chai, Yong Chen, Yuan Yuxing, Guiangjia, Li Zoe Johnson	Device	This bone regeneration product contains stem cells for dense or spongy bone regeneration and may include a growth factor. It treats critical-sized bone defects using 3D-printed scaffolds with HA and TCP or polymer-based scaffolds. Additional components include a growth factor and a Smurf1 inhibitor	Granted: 2022-11-01. Published: 2022-11-01	[129]
11	A kind of artificial bone scaffold composite material and preparation method based on kangaroo bone	CN105536065B (China)	Yan Lamei, Yuan Youwei, Li Li, Jincheng, Li Pengwei, Liu Yi	Drug system	Ingredients: Kangaroo bone powder, calcium pyrophosphate, pore-foaming agent, solvent, catalyst-type curing agent, adhesive blend. Advantages: This composite material addresses mechanical deficiencies while maintaining biocompatibility. It offers improved rigidity, hardness, and elasticity modulus compared to existing PLA/PLGA and PLA/PLGA/TCP scaffolds	Granted: 2018-09-18. Published: 2018-09-18	[130]
12	Stable heterodimeric antibody design with mutations in the fc domain	KR1019 KR101973930B173930B1 (South Korea)	Eric Escobar, Cabrera, Kreudenstein, Thomas Spratter, Ponsurjit Bhimarao, Dixit, Paula Irene, Lario, David Kai, Yuen Funigor, Edmondo, Paolo De Angelo	Drug system	The scaffold enhances selectivity among various Fc receptors, surpassing the natural homodimer (symmetric) Fc molecule. By increasing the variant Fc heterodimer (e.g., CH2 and CH3), it achieves better stability and purity. These novel molecules are designed to modify the natural behavior of antibodies and include complexes of heterogeneous components for therapeutic use	Application granted: 2019-04-29	[131]
13	Method of excision of femoral component of hip joint endoprosthesis	RU2611897C1 (Russia)	Vadim Nikolaevich, Golnik Lyudmila, Grigorievna Grigoricheva, Denis Anatolyevich, Dzhukhaev Alexey, Mikhailovich Ivanyuk, Sergey Anatolyevich, Merkulov Sergey, Vladimirovich Popov Konstantin, Mikhailovich Shkretov	Device	This invention describes a method for removing the femoral component of a hip joint endoprosthesis. It involves drilling a small hole in the femur, creating a guide channel, and using a Gigli saw to cut around the prosthesis, allowing it to be removed from the femoral canal	Application granted: 2017-03-01	[132]

Future perspectives and emerging trends

Recent advancements in materials science, nanotechnology, and imaging modalities are likely to lead to new classes of nanocarriers with enhanced theranostic capabilities [133]. The combination of materials with different properties in the same biocompatible scaffolds is expected to create new applications of great potential for scaffold-based theranostic systems in targeted drug delivery and imaging [134-137]. Scalability, robust imaging, and interactive platforms are all crucial aspects that need to be carefully designed for the successful in vivo implementation of such systems. Moreover, the theranostic concept can also be broadened beyond combined drug delivery and imaging into combined tissue engineering and drug delivery. In particular, scaffolds capable of delivering drugs can be designed for controlled dual release to disturb simultaneously several biological processes [138]. Such systems can find applications for enhancing angiogenesis in critical-sized bone defects together with the inhibition of the bone resorption process by the same scaffold. In parallel, scaffolds can be designed for photo-activated theranostic applications with controlled drug release in combination with imaging capabilities [139-141]. Driven by the abundance of new healthcare data, fast and cost-effective chemical and biological analyses, the growing installed base of devices for healthcare monitoring, and the increasing demand for patient-centric care and patient engagement, technologies enabling decentralized health monitoring are expected to burgeon exponentially in the coming years [142]. This exponential growth can be attributed to a number of factors, including the remarkable advances in wireless sensor networks and high-speed wireless broadband and the miniaturization of sensors, which are collectively paving the way for health monitoring tools to transcend the boundaries of healthcare institutions [143-146].

Indeed, comprehensive personal healthcare monitoring now has the transformative capacity to involve the real-time analysis of multiple analytes in body fluids by means of highly advanced and incredibly compact miniaturized sensors that can operate efficiently outside the confines of traditional clinic settings. Moreover, the modern era of digital connectivity ensures easy access to the vast reservoirs of collected continuous data via a plethora of devices seamlessly connected with the wide-reaching power of the internet [147]. The convergence of five emerging and highly promising fields of technology is expected to further accelerate this critical transition into a remarkable digital personalized health monitoring era. The paramount importance of the first of these fields lies in the imperative need to develop robust biomarker detection and quantification mechanisms by means of personal health monitoring systems that operate autonomously outside the established realm of the clinic or clinical laboratory [148-151]. Interestingly, this incredible shift towards decentralized health monitoring is also being driven by a growing societal desire for active participation in the continuous monitoring of one's own health condition [152]. Empowered by the high prevalence of mobile smartphones and the ever-expanding interest of developers in incorporating healthcare applications, consumers are now increasingly encouraged to take charge of their own well-being and make informed decisions about their health.

Furthermore, the ubiquity of mobile smartphones and widespread internet access has facilitated the rapid integration of healthcare apps into everyday life, thereby enabling individuals to actively engage with their health data and make well-informed decisions regarding their overall well-being [153-158]. This newfound accessibility to healthcare information has revolutionized the way individuals interact with their own health. Moreover, the advent of cloud technology and cloud computing has completely revolutionized the landscape of health monitoring, allowing for seamless data storage, analysis, and sharing. The ability to store vast amounts of health-related data in the cloud has not only made its retrieval more convenient but has also paved the way for groundbreaking research and data analysis, ultimately enhancing patient outcomes [159,160]. Lastly, the need for new and innovative health monitoring devices has emerged as an indispensable aspect of this transformative era. The development of devices capable of detecting and quantifying analytes at the point of need, as opposed to relying solely on sophisticated and often expensive laboratory equipment, has proven to be a game-changer in bringing personalized health monitoring to the masses. This widespread availability of affordable and user-friendly devices has revolutionized healthcare by empowering individuals to monitor their health in the comfort of their own homes [161].

## Conclusions

The confluence of numerous groundbreaking technologies and societal shifts has paved the way for the rapid and transformative evolution of decentralized health monitoring into a digital personalized health monitoring era. The future is bright, as this revolutionary era promises to empower individuals, enhance patient outcomes, and revolutionize the way we approach and interact with our own health and well-being. Driven by the abundance of new healthcare data, fast and cost-effective chemical and biological analyses, the growing installed base of devices for healthcare monitoring, and the increasing demand for patient-centric care and patient engagement, technologies enabling decentralized health monitoring are expected to burgeon. The advances in wireless sensor networks and high-speed wireless broadband and the miniaturization of sensors are expected to provide health monitoring tools outside of healthcare institutions. Comprehensive personal healthcare monitoring thus has the potential to involve the real-time analysis of multiple analytes in abundant body fluids by miniaturized sensors outside of the clinic and easy access to collected continuous data via devices connected with the internet. The convergence of five emerging and promising fields of technology is expected to accelerate this transition into a digital personalized health monitoring era. The advancement of decentralized health monitoring systems is driven by several key factors: the growing need for personal biomarker detection outside clinical settings, increased consumer interest in continuous health monitoring, the widespread use of smartphones with healthcare apps, access to the internet and cloud computing for real-time data management, and the demand for affordable devices capable of point-of-need analyte detection, reducing reliance on specialized laboratory equipment.
